# Evaluation of nano spray drying as a method for drying and formulation of therapeutic peptides and proteins

**DOI:** 10.3389/fphar.2015.00140

**Published:** 2015-07-07

**Authors:** Yusuf A. Haggag, Ahmed M. Faheem

**Affiliations:** ^1^Saad Centre for Pharmacy and Diabetes, School of Pharmacy and Pharmaceutical Sciences, Ulster UniversityColeraine, UK; ^2^Department of Pharmaceutical Technology, Faculty of Pharmacy, University of TantaTanta, Egypt

**Keywords:** spray drying, peptides, proteins, nanoparticles, microparticles

The high sensitivity of peptides and proteins to physicochemical stresses during processing and storage is the major hurdle against its pharmaceutical applications. Peptide-based drug formulations are commonly prepared as solid dosage forms because more stability can be achieved in the solid rather than in the liquid state (Ameri and Maa, 2006). The most common methods to increase the stability of peptides and proteins are drying and the use of stabilizing excipients (Maltesen and van de Weert, 2008). So far, spray drying and freeze drying are the most popular methods of drying peptides and protein solutions in the Pharmaceutical industry. Other drying methods, like spray coating, spray freeze drying (SFD), and supercritical fluid technology and their different modifications, are mostly used on a small scale, especially for research purposes (Franks et al., 1991; Marreto et al., 2006). The optimum choice of drying technique will depend mainly on the economics of drying and on the intended route of drug administration (Webb et al., 2002).

Compared to freeze drying, spray drying is a faster and more economical, single step, drying method which can be designed as a continuous drying process (Masters, 1972). Spray drying is suitable for heat-sensitive materials, despite the high temperatures of the drying gas, owing to the cooling effect of the evaporating solvent which keeps the droplet temperature relatively low (Masters, 1972). Another advantage of spray drying technique is its ability to control the particle size and the morphology of the dried powder by varying the process parameters and the formulation factors (Masters, 1972). Moreover, spray dried powders are commonly prepared when the intended route of administration is *via* inhalation, so that this technique is of prime importance for pulmonary delivery of protein pharmaceuticals (Maaand and Prestrelski, 2000; Lee, 2002). However, low powder yield is still the major drawback in the development of spray dried pharmaceuticals because of small amounts of expensive active ingredients which are available after the early stages of development (Broadhead et al., 1994). Other instability problems encountered when spray drying an aqueous solution of a pure protein, include aggregation and subsequent loss of activity (Adler and Lee, 1999; Tzannis and Prestrelski, 1999). These instabilities can be ameliorated by formulation measures, such as the inclusion of disaccharides or surfactants in the liquid feed to prevent protein aggregation or inactivation, a strategy that can be also implemented to improve storage stability of therapeutic peptides, proteins and heat-sensitive enzymes without great denaturation (Broadhead et al., 1992, 1993; Adler and Lee, 1999). The mechanisms of preferential exclusion, water replacement and glass immobilization are quite similar to those involved in lyophilization to explain the sugar's role in stabilizing protein and peptide molecules during the drying process (Lee, 2002).

During spray drying, the way in which stabilizing excipients react with proteins can control the choice of the additives and the processing conditions to preserve the protein properties, stability, and biological activity. Surfactants such as Cremophor®EL and Pluronic®F-127, and sugars like cyclodextrin and inulin, maintained lysozyme thermal stability after spray drying. Pluronic®F-127 prevented lysozyme aggregation meanwhile preserved its biological activity similar to β-cyclodextrin and inulin which maintained lysozyme biological activity. The improved stability of the spray dried lysozyme using Pluronic®F-127 as a stabilizer revealed the promising delivery of proteins via inhalation and injections (Haj-Ahmad et al., 2013).

It is impartial to conclude that the most common stresses involved during spray drying of different peptides and protein molecules are adsorption, shear stress, thermal stress, and dehydration stress. These stresses can result in unfolding of protein structure with subsequent aggregation and denaturation, thus creating substantial worries about possible loss of their biological activity (Lee, 2002). In addition to the influence of formulation parameters, the extent of protein degradation during spray drying is greatly affected by the spray drying process conditions such as inlet temperature and spray rate, which also have an impact on the particle morphology and the aerodynamic properties of the powder (Cabral-Marques and Almeida, 2009). Therefore, judicious selection of machine size and process parameters allows the preparation of protein loaded inhalable powders characterized by minimal protein damage, suitable residual moisture content, good aerodynamic properties, and satisfactory storage stability (Lee, 2002; Chan, 2003).

Recent developments achieved in the area of particle engineering *via* spray drying in the last two decades were coinciding with the development of different pulmonary therapeutics administered traditionally by injection (Patton and Byron, 2007). The pulmonary route was found to be beneficial for systemic delivery of different proteins and peptides (Johnson, 1997), in particular insulin (Patton et al., 1999). Micro- and nanoparticles fabrication *via* spray drying has been introduced as an attractive manufacturing technique in the pharmaceutical engineering owing to its wide applicability in addition to its contribution to high stability and efficacy of the final particulate dosage form (Vehring, 2008).

The spray drying technology received a great attention for the formulation of micro/nanoparticulate controlled delivery systems (Giunchedi and Conte, 1995; Gavini et al., 2003). It can be used as a rapid and convenient process to prepare microparticles with precise physical morphologies (Alcock et al., 2002). Spray drying has been employed to enhance drug solubility and bioavailability of active ingredients, modified release, and pulmonary delivery of proteins or vaccines (Cal and Sollohub, 2010; Sollohub and Cal, 2010). Recent studies highlighted the potential use of spray drying as an effective technology to deliver macromolecular therapeutics encapsulated in poly (d,l-lactide-co-glycolide) (PLGA) polymer and its derivatives. PLGA has been studied most intensively to encapsulate different types of proteins through spray drying processing. However, the implementation of double emulsification (w/o/w) or phase separation techniques is a prerequisite before the application of the spray drying technique (Bittner et al., 1998; Blanco-Príeto et al., 2000; Giunchedi et al., 2001).

Current progress in the spray drying paved the road to introduce a new advanced technology such as the Nano Spray Dryer B-90, recently developed by BÜCHI Labortechnik AG, which resulted in high particle recovery rates up to milligram sample amounts of powder particles with particle sizes between 300 nm and 5 μm (Figure 1). The Nano Spray Dryer was designed to generate millions of precisely sized tiny droplets every second by using a piezoelectric driven, vibrating membrane in the spray head. The final, dried particles are separated by the aid of an electrostatic particle collector with high product recovery (Li et al., 2010; Bürkia et al., 2011; Heng et al., 2011; Lee et al., 2011; Schmid et al., 2011; Schafrothb et al., 2012).

**Figure 1:**
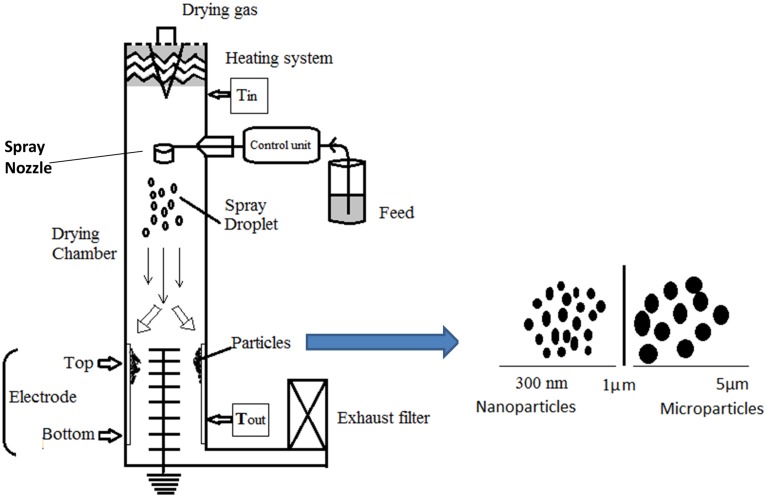
**Schematic design of the Nano Spray Dryer B-90 and its products of nano/microparticles with an average size range of 300 nm–5 μm**.

The main drawbacks of classical spray drying were the high sample volume required (minimum 50 mL), the low yield obtained (maximum 70%), and the big particle size (minimum 2 μM). However, by using the Nano Spray Dryer with its new advanced technology of the spray head, the heating system, and the electrostatic particle collector, the sample amount or volume can be as small as 200 mg or 2 mL, the final product yields increased up to 90%, the particle sizes decreased up to 300 nm with narrow size distribution, and, eventually, fast drying process (up to 150 ml/h) was attained (Li et al., 2010; Bürkia et al., 2011; Heng et al., 2011; Lee et al., 2011; Schmid et al., 2011; Schafrothb et al., 2012).

The major advantage of having nanosize powder, especially in the field of proteomics and genomics, is to enhance the stability and therapeutic potential of these drugs to become more effective and less toxic. The *in vivo* biodistribution and clearance of these drugs mainly depend on their size (Wang et al., 2011). Nanosize is a key parameter that significantly affects cellular and tissue uptake of nanoparticles, as it influences their internalization mechanisms. Submicron size particles are taken up more efficiently compared to the larger size microparticles (Zauner et al., 2001).

This technology is widely used in different pharmaceutical application owing to its unique features of processing the small sample amounts and the high yields produced. It is ideal for the spray drying of precious samples, inhalable drugs as protein dry powder inhalers and stabilization of heat-sensitive vaccines, proteins, and hormones. On the other hand, it can be used for fabrication of liposomes and polymeric nanoparticles (nanocapsules of biodegradable and biocompatible polymers) encapsulating different drugs for controlled drug release and higher bioavailability. Moreover, this technique can be used for drying of various excipients for controlled drug release purposes as hydroxypropyl methylcellulose (HPMC), trehalose, mannitol, and lactose (Li et al., 2010; Bürkia et al., 2011; Heng et al., 2011; Lee et al., 2011; Schmid et al., 2011; Schafrothb et al., 2012).

This novel spray drying approach can be effectively used as an alternative technique for nanoparticle engineering (Li et al., 2010). The Nano Spray Dryer B-90 can be used for a wide variety of applications including spray drying of solutions, nanosuspensions, nanoemulsions, or micro and nanoparticles as well as for structural transformations. Also, it has been employed for the preparation of submicron polymeric particles, the encapsulation of nanoemulsions (Schmid et al., 2011), and the drying of pharmaceutical excipients and models of active ingredients (Heng et al., 2011). Recently, Nano Spray Dryer B-90 has been applied for producing protein nanoparticles (Lee et al., 2011).

Moreover, the optimization of the formulation parameters, like the polymer molecular weight, degradation characteristics and the drug–polymer interactions besides spraying parameters such as spray mesh diameter, spray rate, sample concentration, and sample flow rate had a great impact on the particle size, production yield, encapsulation efficiency, solid state solubility, and controlled release profiles of the engineered nanoparticles (Li et al., 2010).

Previous studies showed that different proteins like bovine serum albumin (BSA) and β-galactosidase could be spray dried without activity loss using the Nano Spray Dryer B-90. Optimization of the process setting like the inlet temperature, the spray mesh size and the ethanol content resulted in respirable size and high yields (approximately 90%) in case of β-galactosidase. The impact of other experimental conditions as BSA solution concentration, stabilizer concentration, drying air flow rate on the aerodynamic properties of protein nanoparticles offered a simple and alternative approach for drug delivery of protein nanoparticles (Bürkia et al., 2011; Lee et al., 2011).

Nano Spray Dryer was used in cost effective production of high yields of vildagliptin nanospheres designed to treat type 2 diabetic patients. These mucoadhesive nanospheres were marked by their narrow particle size distribution of 445 nm. This favorable nano size distribution made it suitable for oral administration (Harsha et al., 2015).

The spray drying of peptides and proteins from aqueous and/or organic liquid feeds can be effectively achieved by using the Nano Spray Dryer. Small therapeutic peptides and proteins, which showed high water solubility, can be efficiently dried from their aqueous liquid feeds in an economical way. Other peptides, which show poor aqueous solubility, are difficult to be dried from their aqueous feeds; therefore spray drying of these peptides dissolved in non-aqueous solvents may be necessary to meet the demands on economical production and good particle formation. Characterization of the spray dried protein regarding the particle morphology, the physical state, the residual content of water and other organic solvent, the glass transition temperature, and the biological activity should be used as criteria for judging successful protein powder formulation.

In addition to the solubility behavior of different peptides and proteins, the behavior of different excipients for protein stabilization and possible stress factors experienced by a protein during spray drying can be controlled to improve the final product. A variety of therapeutic peptides and heat sensitive model enzymes could be successfully spray dried without activity loss using the Nano Spray Dryer when the optimized process settings were adjusted to produce dried protein particles of respirable size in a high yield. Other essential optimization is to identify stabilizers, which are better suited to protect different proteins during the spray drying process with respect to the powder's inhalation properties, the storage stability and the shelf life of the final product.

The major drawbacks are represented when the harsh processing steps render proteins or peptides more susceptible to physical and chemical instability through aggregation, denaturation, oxidation, and cleavage. Proteins are exposed to several shear stresses like shaking, pumping, and nozzle atomization during this spray drying process. Adsorption of proteins and peptides to different interfaces can result in unfolding of their structure that finally leads to the formation of aggregates. However, thermal stress is very important as proteins lose their native structure when exposed to sufficiently elevated temperatures and the native state of a protein is stable in a limited temperature range. Removal of water by dehydration processes, like spray drying, might lead to structural modification and protein denaturation.

However, the pros and cons of Nano Spray Dryer on the level of nanoparticles formulation are quite different. The recovery of small particles intended for inhalation is significantly low using the classic spray dryer because of their poor separation in cyclone collectors owing to their low mass, a major drawback which was successfully overcome by recent developments and progress of nano spray drying technology as discussed earlier. At the nanoparticles' formulation level, the technology was successfully utilized for controlled delivery of peptides and proteins. The technique involves rapid transformation of micro and nano-dispersed droplets into dried particles by atomization of a liquid feed. Nevertheless, a high solid content in the liquid feed is a prerequisite to achieve good particle formation and to minimize the production costs.

Nano Spray Dryer is a very fast, easy and cost-effective powder generating device, which can be used to prepare powders in dry forms with high yields. Owing to its simplicity as a one step process, the emulsification/spray drying method has been used as an alternative to double emulsification method for the fabrication of PLGA micro and nanospheres containing water-soluble drugs. Moreover, this spray drying method has an additional advantage in the preparation of sterile injectable particulate delivery systems because it can be conducted in an aseptic condition, as the device could be easily cleaned and sterilized and the atomized air could be filtered. On the other hand, the incorporation of stabilizers such as poly (vinyl alcohol) and other amphiphilic surfactants, which are difficult to remove, is not recommended to minimize the preparation efforts and to prevent the side-effects from the residual surface located surfactant.

The hydrophobic ion pairing (HIP) technique based upon spray drying can be employed as a simple process to improve hydrophilic peptide encapsulation in lipophilic carriers to form micro and nanoparticles. These micro and nanoparticles can modify the release of the encapsulated peptides while retaining the biological activity (Alcock et al., 2002).

Combination of (HIP) method with organic spray drying and emulsion solvent diffusion method enhanced insulin encapsulation and release from PLGA nanoparticles. These nanoparticles enhanced the oral bioavailability of insulin thereby it was promising for oral insulin delivery (Sun et al., 2015).

While PLGA nanoparticles encapsulating different drugs as peptides, proteins and small molecules can be produced with controlled physicochemical properties using Nano Spray Dryer, the ability to process small volumes to produce a high yield of small particles still represents the major advantage of this relatively new approach.

## Conflict of interest statement

The authors declare that the research was conducted in the absence of any commercial or financial relationships that could be construed as a potential conflict of interest.
